# The Changing Use of Intravenous Opioids in an Emergency Department

**DOI:** 10.5811/westjem.2015.10.28454

**Published:** 2015-12-14

**Authors:** Mark E. Sutter, Garen J. Wintemute, Samuel O. Clarke, Bailey M. Roche, James A. Chenoweth, Rory Gutierrez, Timothy E. Albertson

**Affiliations:** * University of California, Davis, Department of Emergency Medicine, Sacramento, California; † University of California, Davis, Department of Emergency Medicine, Division of Medical Toxicology, Sacramento, California; ‡ VA Northern California Health Care System, Mather, California; § University of California, Davis, Department of Pharmacy, Sacramento, California; ¶ University of California, Davis, Department of Internal Medicine, Sacramento, California

## Abstract

**Introduction:**

Government agencies are increasingly emphasizing opioid safety in hospitals. In 2012, the Joint Commission on Accreditation of Healthcare Organizations (JCAHO) started a sentinel event program, the “Safe Use of Opioids in Hospitals.” We sought to determine if opioid use patterns in our emergency department (ED) changed from 2011, before the program began, to 2013, after start of the program.

**Methods:**

This was a retrospective study of all adult ED patients who received an intravenous opioid and had a serum creatinine measured. We recorded opioids used, dose prescribed, and serum creatinine. As an index of the safety of opioids, uses of naloxone after administration of an opioid was recorded.

**Results:**

Morphine is still the most commonly used opioid by doses given, but its percentage of opioids used decreased from 68.9% in 2011 to 52.8% in 2013. During the same period, use of hydromorphone increased from 27.5% to 42.9%, while the use of fentanyl changed little (3.6% to 4.3%). Naloxone administration was rare after an opioid had been given. Opioids were not dosed in an equipotent manner.

**Conclusion:**

The use of hydromorphone in our ED increased by 56% (absolute increase of 15.4%), while the use of morphine decreased by 30.5% (absolute decrease 16.1%) of total opioid use from 2011 to 2013. The JCAHO program likely was at least indirectly responsible for this change in relative dosing of the opioids. Based on frequency of naloxone administered after administration of an opioid, the use of opioids was safe.

## INTRODUCTION

Preventing adverse medication events is a high priority for healthcare providers, hospitals, and governmental agencies. In 2012, the Joint Commission on Accreditation of Healthcare Organizations (JCAHO) issued a sentinel event-alert program, “Safe Use of Opioids in Hospitals.”^1^ Since the start of the program, governmental and regulatory agencies have closely evaluated the use of opioids when assessing hospitals. One area of interest has been the use of morphine in patients with renal dysfunction. Morphine has two major metabolites: morphine-6-glucuronide and morphine-3-glucuronide.^2^ Morphine-6-glucuronide can accumulate in patients with renal dysfunction, leading to respiratory depression and failure.^2^ With heightened awareness of this risk, our hospital has, through various measures, such as pharmacy-order verification and intervention, limited the prescribing of morphine for patients with renal dysfunction in the emergency department (ED).

In intensive care settings, participation of dedicated pharmacy services has reduced medication errors.^3^,^4^ While such services have been less studied in EDs, pharmacists are becoming more involved in emergency care, assisting with medication reconciliation and order verification.^5^ One area in which pharmacists are intervening is in suggesting alternative opioids for morphine in patients with renal dysfunction. Intravenous opioid alternatives include hydromorphone and fentanyl; hydromorphone is the more commonly used opioid because the pharmacokinetics of fentanyl are shorter acting.^6^,^7^

In addition to recognizing the importance of using opioid replacement for morphine in patients with renal dysfunction, it must be appreciated that opioid medications should be dosed in equipotent amounts. In the era of electronic medical recordkeeping, dosing choices often are pre-selected, or an option is given to manually input a desired dose. About the time the “Safe Use of Opioids in Hospitals” program was begun, the makers of hydromorphone reduced the recommended dosing range from 0.2–2mg to 0.2–1mg.^8^ Despite the manufacturer’s recommendation, however, our institution did not change our pre-selected doses until 2014.

Given that around the start of 2012, JCAHO instituted the “Safe Use of Opioids in Hospitals” program, and the manufacturer of hydromorphone reduced its recommended dosing, we have examined whether these measures affected our usage of opioids in the ED. We compared opioid usage in the year 2011, before publication of the advisories on opioid use, to usage in 2013. Our aims were to determine if there was a change in which opioids were used, to determine if the medications were dosed in an equipotent manner, and to determine if there was a change in opioid-related adverse events, as defined by the use of naloxone after an opioid was given.

## METHODS

This retrospective study was performed in an academic, urban, tertiary care, Level I trauma center which has an emergency medicine training program. The department has an annual census of approximately 85,000. In part one of the study, data was abstracted from the first 35,000 subjects seen each year (2011 and 2013) who met all inclusion criteria. The inclusion criteria were the following: patients aged 18 years or older; parenteral opioid administered during the ED visit; and serum creatinine measured during the visit. The intravenous opioids studied were morphine, fentanyl, and hydromorphone. If subjects met inclusion criteria we collected the following data: opioid used, opioid dose, serum creatinine, and, if available, weight. In the second part of the study, all ED use of naloxone during 2011 and 2013 was evaluated. We aimed to quantify how frequently naloxone was used after an opioid was given and if any particular opioid was associated with increased naloxone use.

Initial statistical evaluation of the data included a student’s t-test to evaluate for statistical significance. Despite log transformation of the data due to the non-normal distribution, every result reached statistical significance, despite no clinical difference in the data, due to the large size of the data being analyzed. We therefore chose to present the data in the form of medians with interquartile ranges. This study was approved by our institutional review board.

## RESULTS

Composite data and opioid-specific data are summarized in Tables 1a, 1b, and 1c. In composite data it can be seen that the total doses of opioid administered and the doses per patient were moderately higher in 2013 than in 2011. Patients’ median serum creatinine concentrations in the two years were not significantly different. Recorded weights were available in only 3% of the subjects, so this measurement was excluded from further analysis.

In opioid-specific data, no significant differences between the years 2011 and 2013 in patients’ ages, dose per administration of opioid, or medium serum creatinine concentrations were recorded for patients who received fentanyl, hydromorphone, or morphine.

We assessed the equivalency values for the use of hydromorphone and fentanyl compared with morphine in 2011 and 2013. In both years, we found that the opioids were not prescribed in a dose-equivalent manner. That is, the usual dose of hydromorphone prescribed (1mg) was equivalent to 7mg of morphine, whereas the usual dose of morphine prescribed was 4 mg. The usual dose of fentanyl prescribed (50μg) was equivalent to 5mg of morphine.^9^

The use of naloxone is presented in Tables 2a and 2b. The rate of usage of naloxone was similar in 2011 and 2013. No differences between 2011 and 2013 in the patients’ ages or serum creatinine concentration, or in doses of naloxone given were found. In 16 of the 22 patients given naloxone after administration of an opioid, the patients’ home medication lists included sedative hypnotics, including benzodiazepines; sleep medications; or medications classified as “muscle relaxants.” Additionally, three of the patients had ethanol levels of 120mg/dL, 156mg/dL, and 180mg/dL, respectively. No single opioid (fentanyl, hydromorphone, or morphine) was associated with uniquely higher rates of naloxone usage. Only a small percentage of patients who received naloxone received it after the administration of an opioid in our department (about 2%); the vast majority of naloxone was given for diagnostic purposes.

## DISCUSSION

The principal aim of this study was to determine if JCAHO’s “Safe Use of Opioids in Hospitals” program and the manufacturer’s reduced recommended dosage of hydromorphone (both instituted in about early 2012) influenced the prescribing practices for opioids in our ED. We sought to determine if there was a change in which opioids were used, if the medications were dosed in an equipotent manner, and if there was an increase in opioid-related adverse events, as defined by the use of naloxone after receiving an opioid in the ED.

We found, first, a modest increase in the percentage of hydromorphone prescribed, with a corresponding decrease in the percentage of morphine prescribed. The explanation for this change is not definitely known. However, we believe that JCAHO’s program was at least indirectly responsible: The program drew attention to the risks of morphine in patients with renal dysfunction, and our pharmacy responded by emphasizing this risk and in limiting the prescribing of morphine in such patients. Our prescribers also increasingly perceived hydromorphone as generally safer than morphine, at least in patients with renal disease. Whether this perception is justified, however, is not definite since hydromorphone, like morphine, has renally cleared metabolites. Hydromorphone-3-glucoronide, the principle active metabolite of hydromorphone, is renally cleared, and dose reductions for hydromorphone in patients with renal failure also are recommended.^9^,^10^ Hydromorphone-3-glucoronide has been associated also with neuroexcitatory behavior, such as seizures, which occur as often or more often with morphine metabolites.^10^ One potential advantage of hydromorphone is that hydromorphone-3-glucoronide is not associated with respiratory depression mediated by the mu-2 receptor, whereas morphine-6-glucoronide is.^11^ The safest opioid in renal failure is fentanyl, which has no renally excreted metabolites,^11^ but fentanyl is not favored in ED use because of its short duration of action.^6^

Our second major finding was that during the study period we did not prescribe opioids in an equipotent manner; the median dose of hydromorphone was almost double that of the median morphine dose, and fentanyl also was used at a somewhat higher dose than morphine. When evaluating our interquartile ranges, our study showed that we typically gave a dose of 4mg of morphine and 1mg of hydromorphone, which is equivalent to 7mg of morphine. This practice did not change between 2011 and 2013, perhaps because our institution did not update the pre-selected dosing choices in the electronic prescribing system. As institutions set up and modify electronic order set options, attention should focus on dose equivalency since providers often default to pre-selected order entry. At our institution, there also appears to be a belief that hydromorphone has superior analgesic effect, but our data suggest that patients are receiving a significantly more potent doses of hydromorphone than of morphine, and this difference may account for a difference in observed analgesic effect. With the varying complexities of opioid metabolism in high-risk patients, such as those with renal failure, it is important that educational programs, such as ours, adjust appropriately to changing policies for opioid prescribing.

Our third major finding was that naloxone was infrequently needed after opioid use in the ED. Also, no association of a specific opioid with naloxone use was identified. About 98% of naloxone used in our ED was given as a diagnostic or therapeutic aid prior to any opioid given, not in response to suspected adverse reaction to the opioid we had given. Several patients who required naloxone had sedative hypnotic medications listed on their daily medication list, so we suspect that they may have had pre-admission use of agents that could have potentiated the effects of the opioids we administered. However, we did not abstract the medication lists of those patients who received opioids but did not require naloxone, so we cannot draw conclusions about risk factors for naloxone use in our population. The very low frequency of naloxone use after administration of an opioid in our series is evidence that the use of opioids in our department is safe. Of note, we defined in this study that the use of naloxone after an opioid represented an adverse drug event. However, this may not represent a clinical error. Several examples of this were noted during chart abstraction such as a patient with abdominal pain receives an opioid early in their course only to become septic from their intra-abdominal infection and become somnolent. Providers recognizing they treated with an opioid gave naloxone to see if clinical improvement occurred. This would be considered appropriate clinical care, but represented an adverse drug event as defined by our study.

Our final major finding was that total opioid doses increased from 2011 to 2013 at 79,879 to 86,800 doses respectively. This represented a change from 2.28 doses per patient to 2.48 doses per patient of a parenteral opioid. In the era of increasing opioid addition and awareness of opioids, this is a trend that should be further examined.^13^ However, this is likely multi-factorial and other reasons such as ED crowding and longer wait times prior to being treated by a healthcare provider should be considered. Regardless the cause, physicians need heighted awareness of opioid use and prescribing patterns.

## LIMITATIONS

Our study has limitations. Our inability to collect weights on a significant proportion of our patients prevented us from calculating weight-bases dosing of opioids prescribed. Second, this analysis did not include patients who did not have an objective measurement of renal function, so the results cannot be extrapolated to all patients in the ED. Third, because of the large amount of our data it was not feasible to extract the medical records and medication lists of all patients included in the study; rather, we extracted data from the electronic medical record with the help of our Institutional Clinical Translational Science Center, where coding errors could have occurred.

## CONCLUSION

We concluded that, in our ED, the percentage use of hydromorphone increased, while the percentage use of morphine decreased, between the year 2011 and 2013. Although not proven, this change may have resulted from the effects of JCAHO’s program on “Safe Use of Opioids in Hospitals” and opioid-prescribing policies instituted within our medical center. We found also that during the period of the study we had not prescribed opioids on an equipotent basis with morphine. Education on equipotent dosing of opioids is important, as adverse events due to these errors are preventable. Finally, naloxone was infrequently needed after administration of an opioid, a finding that suggests that opioids are used safely in our ED.

## Figures and Tables

**Table 1a:** Demographics and composite data in study of opioid administration.

	2011	2013
Total patients (N)	35,000	35,000
Male	18,812 (45.1%)	19,155 (54.6%)
Total doses administered	79,879	86,800
Fentanyl	2,855 (3.6%)	3,728 (4.3%)
Hydromorphone	21,950 (27.5%)	37,269 (42.9%)
Morphine	55,074 (68.9%)	45,803 (52.8%)
Doses/patient	2.28	2.48

**Table 1b:** Demographics and composite data in study of opioid administration.

	Median	IQR (25–75%)	Median	IQR (25–75%)
Age (yrs)	44.8	31.5–57.3	45.5	32.0–58.1
Creatinine (mg/dL)	0.81	0.63–1.08	0.91	0.60–1.02

**Table 1c:** Opioid-specific data.

	Median	IQR (25–75%)	Median	IQR (25–75%)
Fentanyl				
Age (yrs)	44.9	30.1–56.9	43.9	31.8–58.1
Dose/administration (μg)	50	30.0–100.0	50	40.0–100.0
Creatinine (mg/dL)	0.83	0.72–1.14	0.76	0.60–1.04
Hydromorphone				
Age (yrs)	43.2	31.2–53.6	42.2	33.3–55.2
Dose/administration (mg)	1.0	1.0–1.0	1.0	0.6–1.0
Creatinine (mg/dL)	0.79	0.64–0.99	1.0	0.59–1.05
Morphine				
Age (yrs)	44.2	31.1–56.9	43.0	31.2–57.4
Dose/administration (mg)	4.0	4.0–4.0	4.0	4.0–4.0
Creatinine (mg/dL)	0.82	0.68–1.00	0.86	0.61–1.00

**Table 2a:** Summary of naloxone use in the emergency department.

	2011	2013
Total patients who received naloxone (N)	537	598
Male	202 (37.6%)	246 (41.2%)
Patients given naloxone after opioid was given	10	12

**Table 2b:** Summary of naloxone use in the emergency department.

	Median	IQR (25–75%)	Median	IQR (25–75%)
Age (yrs)	49.6	36.1–56.0	48.6	35.2–55.2
Dose (mg)	0.4	0.4–1.0	0.4	0.2–1.0
Creatinine (mg/dL)	0.93	0.8–0.96	0.94	0.73–1.51
